# In Situ Synchrotron X-ray Diffraction Studies of Hydrogen-Desorption Properties of 2LiBH_4_–Mg_2_FeH_6_ Composite

**DOI:** 10.3390/molecules26164853

**Published:** 2021-08-11

**Authors:** Mohammad R. Ghaani, Michele Catti, Niall J. English

**Affiliations:** 1 Dipartimento di Scienza dei Materiali, Università di Milano Bicocca, via R. Cozzi 53, I-20125 Milano, Italy; michele.catti@unimib.it; 2 School of Chemical and Bioprocess Engineering, University College Dublin, Belfield, D04V1W8 4 Dublin, Ireland

**Keywords:** hydride composite, hydrogen storage, dehydriding reaction, solid–gas reactions, thermodynamic driving force, dehydrogenation

## Abstract

Adding a secondary complex metal hydride can either kinetically or thermodynamically facilitate dehydrogenation reactions. Adding Mg_2_FeH_6_ to LiBH_4_ is energetically favoured, since FeB and MgB_2_ are formed as stable intermediate compounds during dehydrogenation reactions. Such “hydride destabilisation” enhances H_2_-release thermodynamics from H_2_-storage materials. Samples of the LiBH_4_ and Mg_2_FeH_6_ with a 2:1 molar ratio were mixed and decomposed under three different conditions (dynamic decomposition under vacuum, dynamic decomposition under a hydrogen atmosphere, and isothermal decomposition). In situ synchrotron X-ray diffraction results revealed the influence of decomposition conditions on the selected reaction path. Dynamic decomposition of Mg_2_FeH_6_–LiBH_4_ under vacuum, or isothermal decomposition at low temperatures, was found to induce pure decomposition of LiBH_4_, whilst mixed decomposition of LiBH_4_ + Mg and formation of MgB_2_ were achieved via high-temperature isothermal dehydrogenation.

## 1. Introduction

Within the collection of potential candidate materials for solid-state hydrogen storage, metal hydrides—including light metal borohydrides in particular—present themselves as potentially attractive possibilities, and all have seen rather active study [1,2,3]. Metal hydrides are characterised by relatively modest H_2_ yields, typically evincing convincing levels of reversibility in hydrogen exchange. Conversely, borohydrides boast generally high H_2_ content; for the most part, dehydrogenating borohydrides does not lend itself particularly to reversibility.

Since the 1980s more than 80 compounds of complex transition metal hydrides have been identified [4], such as Mg_2_FeH_6_, Mg_2_CoH_5_, and Mg_2_NiH_4_. In these compounds, Mg^2+^ cations paired with an anion complex in the structure (octahedral [FeH_6_]^4−^, square-pyramidal [CoH_5_]^4−^ and tetrahedral [NiH_4_]^4−^) [4]. Mg_2_FeH_6_ contains a large volume of hydrogen, with 150 g L^−1^ volumetric and 5.5 wt% gravimetric hydrogen capacity, and with 77 kJ mol^−1^ as the dehydrogenation enthalpy [5]. The excellent cycle stability of Mg_2_FeH_6_ as well as low-price precursor metals make this compound a good candidate for high-temperature heat storage applications [6].

A rather universal challenge in the case of both hydride families is their greater levels of thermodynamic stability than necessarily wanted, primarily due to their substantial associated heats of dissociation; thus, there is H_2_ release at relatively elevated temperatures, bringing about substantial technical challenges in terms of application. In a sense, this challenge has been tackled by trying to stabilise the hydride’s dehydrogenation products, therefore diminishing the heat of reaction [7]. Upon reacting various hydrides with one another, this may be realised if mixed compounds with good stability are realised via dehydrogenation. In 1968, Reilly and Wiswall [8] developed the idea of using some alloys in reaction with hydrides to make the system less thermodynamically stable through the formation of a new compound with a lower energy level. Concerning the role of enthalpy and entropy on the level of Gibbs free energy (ΔG = ΔH−TΔS), either lowering the enthalpy or increasing the entropy of the reaction can result in a lower ΔG value of the reaction. This change can be visualized using a van ’t Hoff plot, in which the change in the slope reflects the change in enthalpy, and a higher intercept shows a reaction with higher entropy [9]. Indeed, computational modelling shows that this constitutes a new avenue for enhancing the capacity of candidate materials for storing H_2_ [10].

Against this background, we studied 2LiBH_4_–Mg_2_FeH_6_ dehydrogenation from a thermodynamic perspective [11,12]. Interestingly, this mixes the elevated gravimetric hydrogen density of LiBH_4_ (13.9%) [13] with its impressive volumetric counterpart of Mg_2_FeH_6_ (150 g L^−1^) [5]. Other studies on related compounds have also shown similarly great levels of promise [14,15,16,17]. In a study by Li et Al. on a composite of Mg_2_FeH_6_ and LiBH_4_ in different compositions (Mg_2_FeH_6_/LiBH_4_ molar ratios (X) of 0.25, 0.5, and 0.75), the authors reported X = 0.5 as the correct stoichiometric ratio for Mg_2_FeH_6_ and LiBH_4_ joint decomposition [18].

**Figure 1:**
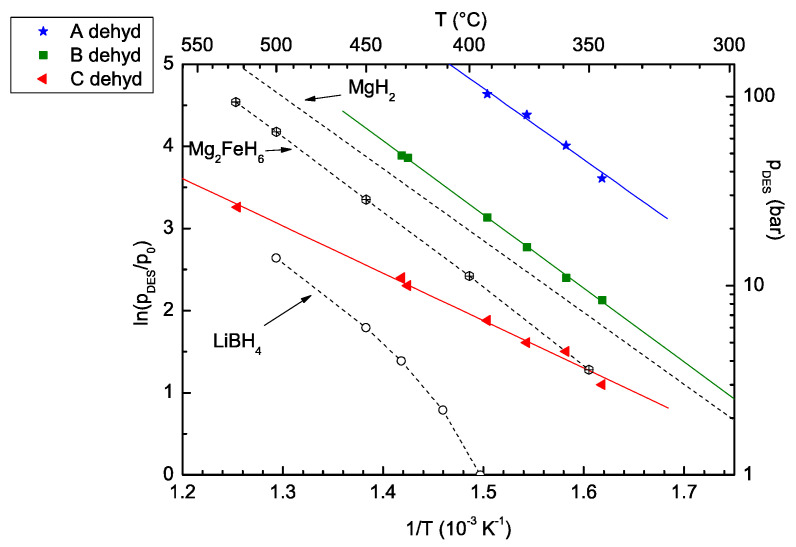
Van ’t Hoff plots of the 2LiBH_4_–Mg_2_FeH_6_ composite’s three-step dehydrogenation reactions (A, B, and C) (full symbols including best fit lines). Open symbols represent literature data for the decomposition of each individual compound—LiBH_4_ (circles) [19] and Mg_2_FeH_6_ (squares) [5]—while dotted lines denote the MgH_2_ dehydrogenation data [20]. Figure adapted, with permission, from [11].

Our previous study’s principal result [11] highlights how dehydrogenation takes place over three distinct steps, upon decrease in pressure under isothermal conditions, or temperature increase in an isobaric system. The thermodynamic condition of each reaction is summarized in Figure 1, and discussed in detail in [11]. Reaction “A” occurs at a lower temperature and higher pressure in comparison with pure Mg_2_FeH_6_ and LiBH_4_, which shows the destabilisation via the joint decomposition reaction. The dehydrogenation reaction paths of reactions “A”, “B”, and “C” were identified based on ex situ XRD analysis of the formed compounds after each step through isothermal decomposition.
**Name****Reaction****Equation**A$2{\mathrm{LiBH}}_{4}\;\left(s\right)+2{\mathrm{Mg}}_{2}{\mathrm{FeH}}_{6}\;\left(s\right)2\mathrm{LiH}\;\left(s\right)+4{\mathrm{MgH}}_{2}\;\left(s\right)+2\mathrm{FeB}\;\left(s\right)+5H_{2}\;\left(g\right)$(1)B${\mathrm{MgH}}_{2}\;\left(s\right)\;\mathrm{Mg}\;\left(s\right)+{\;H}_{2}\;\left(g\right)$(2)C$2{\mathrm{LiBH}}_{4}\;\left(s\right)+\;\mathrm{Mg}\;\left(s\right)2\mathrm{LiH}\;\left(s\right)+{\;\mathrm{MgB}}_{2}\;\left(s\right)+3H_{2}\;\left(g\right).$(3)

To date, the reported reaction mechanism of joint 2LiBH_4_–Mg_2_FeH_6_ decomposition has been investigated under isothermal conditions, and the results have been reported by several groups [11,12,14,15,16,17,18], while the knowledge of the actual reaction path for decomposition under dynamic heating of the mixture, and the present competition between different possible decomposition reactions, is limited. Indeed, the current study’s goal lies in investigating the dehydrogenation of the 2LiBH_4_–Mg_2_FeH_6_ assemblage mechanism in detail, following the applied reaction conditions such as dehydrogenation under vacuum, reaction with temperature-programmed dehydrogenation (dynamic decomposition), and isothermal dehydrogenation using in situ X-ray diffraction data. Moreover, this study presents some evidence on the required conditions for the formation of MgB_2_, and its important role in the recyclability of Mg/LiBH_4_ systems.

## 2. Results and Discussion

### 2.1. Purity of Mg_2_FeH_6_

**Figure 2:**
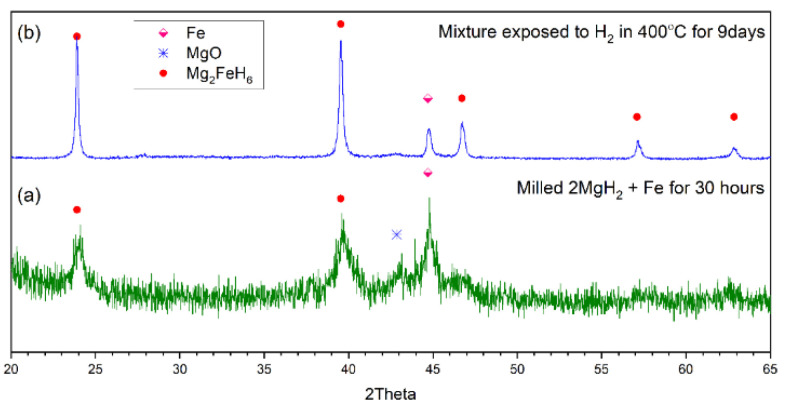
XRD pattern (Cu K-α) of the sample (**a**) milled for 1 day and 6 h, and (**b**) exposed to hydrogen at 400 °C for 9 days.

X-ray measurements of ball-milled powder of 2Mg+αFe (Figure 2a) demonstrated unambiguously the exclusive presence of Mg_2_FeH_6_ and α-Fe, with no evidence of remaining MgH_2_ or Mg. The direct formation of Mg_2_FeH_6_ during the milling process was also observed previously by Huot [21] and Bassetti et al. [22]. Since the MgH_2_ was the only source of hydrogen in the milling vial, the formation of Mg_2_FeH_6_ needs to be followed by the decomposition of MgH_2_ to Mg and free hydrogen molecules, which can occur due to the catalytic effect of Fe on the dehydrogenation of MgH_2_. This effect was previously studied by Bassetti et al. [22] by mixing different concentration values of Fe with MgH_2_ via ball milling to explore its catalytic effect. They concluded that the optimum catalyst (Fe) concentration for MgH_2_ dehydrogenation reactions was around 10 wt%, and lower values seemed to be insufficient to avoid the presence of poorly catalysed regions [22].

Following the hydrogenation step in the Sievert apparatus, just Mg_2_FeH_6_ and a continually diminishing amount of α-Fe was found by X-ray analysis (cf. Figure 2b). During the hydrogenation reaction, the level of iron depletion from the 1- to the 4-day run was evident, but then declined very slowly to a plateau level after 9 days. This suggests that the 4-day high-temperature treatment may also be sufficient for Mg_2_FeH_6_ synthesis. The small amount of MgO present is the result of magnesium oxidation during the milling process [23,24]. The magnesium particles formed during the ball milling have more active surfaces, which can react with the residual oxygen in the glovebox, or with the adsorbed oxygen during the handling process.

### 2.2. Joint Decomposition of 2LiBH_4_–Mg_2_FeH_6_

To evaluate the role of reaction conditions on the selected mechanism, the joint decomposition of 2LiBH_4_–Mg_2_FeH_6_ was studied under three different temperature/pressure conditions: dynamic decomposition under vacuum, dynamic decomposition under 10 bar of hydrogen, and isothermal decomposition.

**Figure 3:**
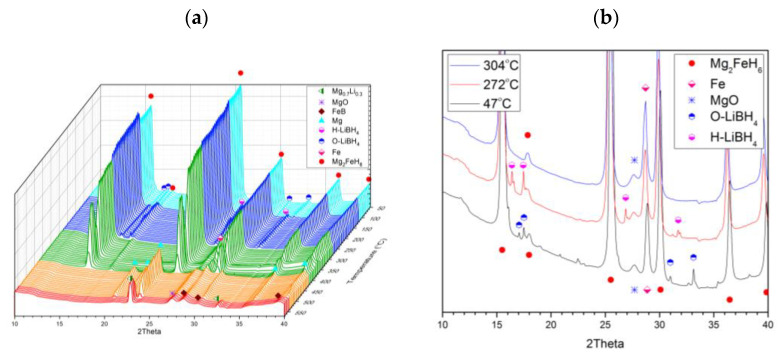
(**a**) In situ synchrotron XRD patterns of 2LiBH_4_–Mg_2_FeH_6_ in dissociation under vacuum (5 °C/min heating). Performed at Lund (λ = 0.99242 Å). (**b**) Phase change and melting of LiBH_4_.

For the first scenario, the evolution of the recorded XRD patterns for 2LiBH_4_–Mg_2_FeH_6_ during its dissociation under vacuum (heat rate 5 °C/min) is presented in Figure 3a. Lithium borohydride was found to act alone and have a transition from an orthorhombic to a hexagonal structure at 110 °C, and melts at 285 °C, followed by dissipation of the LiBH_4_ diffraction peaks in the recorded XRD patterns (Figure 3b). This behaviour is consistent with pure LiBH_4_, as previously reported by Davis et al. [25].

**Figure 4:**
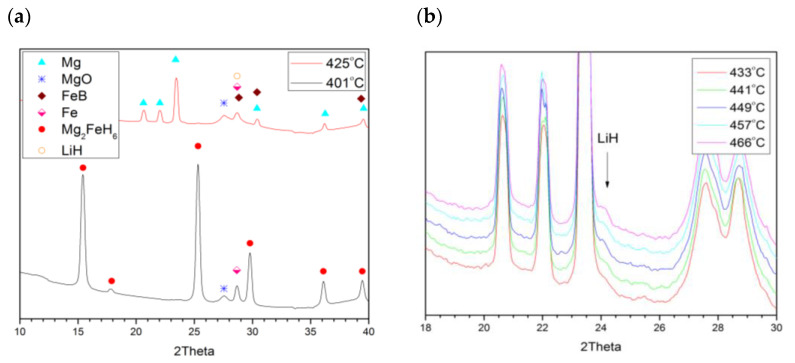
(**a**) Simultaneous A and B reactions (${\mathrm{Mg}}_{2}{\mathrm{FeH}}_{6}+{\mathrm{LiBH}}_{4}\to \mathrm{FeB}+2\mathrm{Mg}+\mathrm{LiH}+9/2H_{2}$). (**b**) LiH formation due to LiBH4 dissociation at 440 °C.

Under vacuum conditions, the first step of Mg_2_FeH_6_–2LiBH_4_ decomposition occurs between 420 and 425 °C (Equation (4)), leading to the production of Mg and FeB (Figure 4a) due to strong heating and the substantial ”driving force” afforded by the vacuum for dehydrogenation. Walker et al. outlined LiBH_4_ dissociation when exposed to Mg in a vacuum, with dynamic variation [9]. It is worth mentioning that Equation (4) is balanced assuming the full conversion of Mg in Mg_2_FeH_6_ to Mg_0.7_Li_0.3_, since no other magnesium-containing phase was identified in the final diffraction pattern. No boron diffraction peak can be observed either, due to the low crystallinity of this product [19]. In the second step of decomposition, at 440 °C, residual LiBH_4_ dissociated to LiH and B, followed by H_2_ release, which is shown clearly in Figure 4b, by the formation of LiH arising from LiBH_4_ dissociation (Equation (5)).

**Figure 5:**
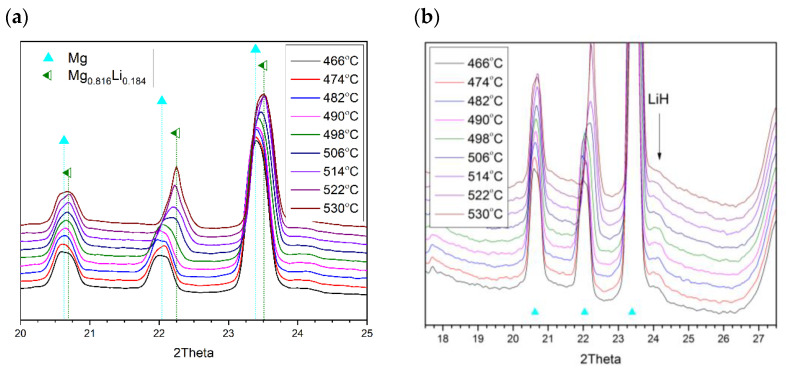
Formation of the Mg_0.816_Li_0.184_ alloy via the reaction of lithium hydride with magnesium: (**a**) Mg_0.816_Li_0.18_ peaks’ appearance; (**b**) LiH consumption.

Lithium hydride formed upon LiBH_4_ dissociation, reacting with Mg at 500 °C, producing the Mg_0.816_Li_0.184_ alloy as shown in Equation (6). From substitutional replacement by Li in Mg, the HCP material’s double-Bragg-peak structure (2θ = 22.02 & 23.4°) moved to larger 2θ values (cf. Figure 5a). This production of an alloy is consistent with earlier investigation of this 2MgH_2_–LiBH_4_ system [9].

**Figure 6:**
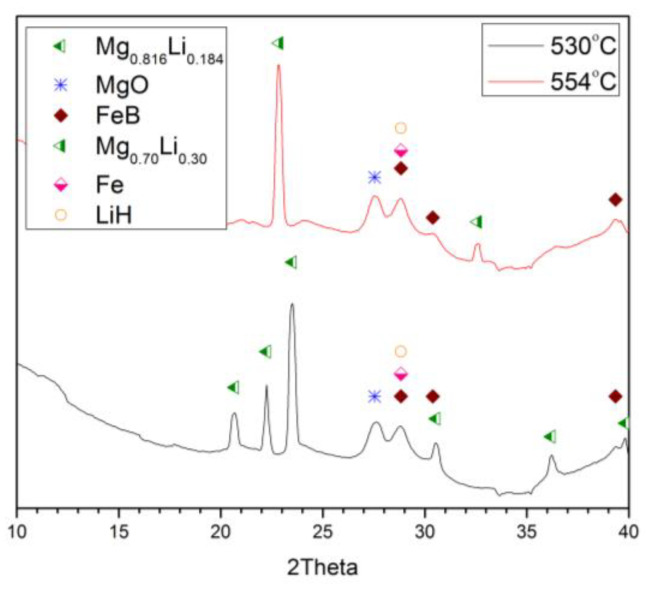
Transformation of Mg_0.816_Li_0.184_ into Mg_0.70_Li_0.30_.

The alloy-formation process serves to diminish the intensity of the lithium hydride peak, with Li penetration into the magnesium lattice (Figure 5b), and then the higher lithium content alloy Mg_0.70_Li_0.30_ begins to form (Equation (7)); here, the two alloys coexist across a temperature range up to the point of the presence of only Mg_0.70_Li_0.30_ (Figure 6). It ought to be noted that in the final case (at 586 °C), the lithium hydride spectral signature remains, and the peak area associated with the alloy remains essentially unaltered. In such a way, it may be ascertained that the dissociation of 2LiBH_4_–Mg_2_FeH_6_ under vacuum, with a predetermined thermal rate (5 °C/min), proceeds through Equation (8).
**Step****Reaction****Temperature (°C)****Equation**1${\mathrm{LiBH}}_{4}+{\;\mathrm{Mg}}_{2}{\mathrm{FeH}}_{6}\to \mathrm{FeB}\;+\;\mathrm{LiH}\;+2\mathrm{Mg}\;+4.5H_{2}$425(4)2${\mathrm{LiBH}}_{4}\to \mathrm{LiH}\;+\;B+1.5H_{2}$440(5)3$2\mathrm{Mg}\;+2\mathrm{LiH}\to 2.45{\mathrm{Mg}}_{0.816}{\mathrm{Li}}_{0.184}+1.55\mathrm{LiH}+0.225H_{2}$500(6)4$2.45{\mathrm{Mg}}_{0.816}{\mathrm{Li}}_{0.184}+1.55\mathrm{LiH}\to 2.86{\mathrm{Mg}}_{0.7}{\mathrm{Li}}_{0.3}+1.14\mathrm{LiH}+0.205H_{2}$+554(7)$2{\mathrm{LiBH}}_{4}+{\;\mathrm{Mg}}_{2}{\mathrm{FeH}}_{6}\to \mathrm{FeB}+1.14\mathrm{LiH}+2.86{\mathrm{Mg}}_{0.7}{\mathrm{Li}}_{0.3}+B+6.43H_{2}$(8)

**Figure 7:**
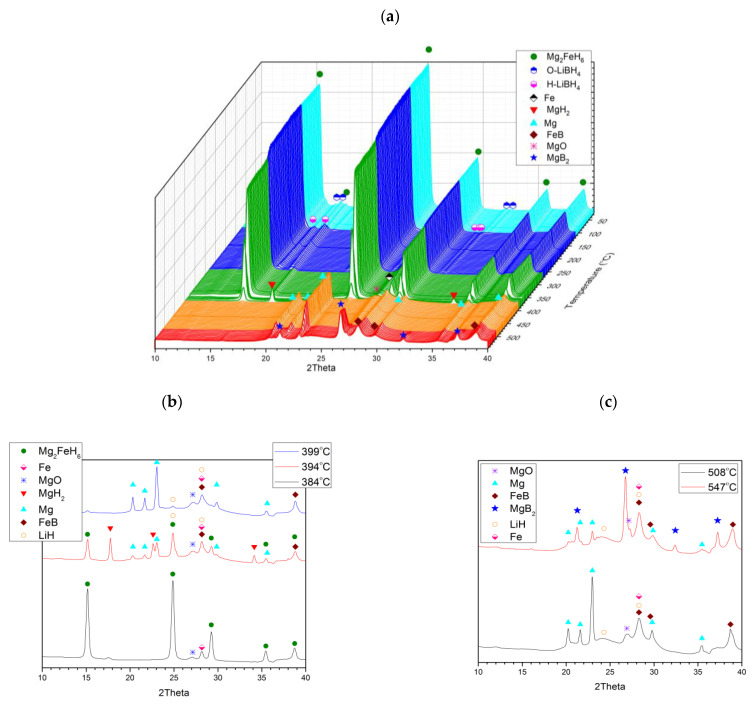
(**a**) In situ synchrotron XRD patterns of 2LiBH_4_–Mg_2_FeH_6_ dissociation (heat rate 5 °C/min) in a 10-bar hydrogen atmosphere. Measured at Lund (λ = 0.99242 Å). (**b**) Reactions A and B at different temperatures. (**c**) Dissociation of LiBH_4_ with concomitant consumption of magnesium to form MgB_2_ (the C substep).

As the second decomposition condition, dynamic decomposition of this mixture under 10 bar of H_2_ pressure was also tracked in real time, using the same diffraction method. Rather different findings were gleaned (Figure 7a), given that all three different (A, B, C; Equations (1)–(3), respectively) reactions were found to take place at differing temperatures, with the ultimate dissociation end-products being FeB and MgB_2_, alongside residual magnesium. For the substeps of reactions A and B, they are only partially resolved by temperature. Figure 7b shows for the 394 °C pattern that the products of reactions A and B are both present. Compared to vacuum decomposition, MgH_2′_s dissociation kinetics are more sluggish due to the slight overpressure, as opposed to underpressure. Upon heating, the reaction C substep occurred at 510 °C, and MgB_2_ was detected via the reaction of magnesium with liquid LiBH_4_. (Figure 7c). This observation is consistent with expectations of the reaction from our ex situ XRD readings [11,12].

**Figure 8:**
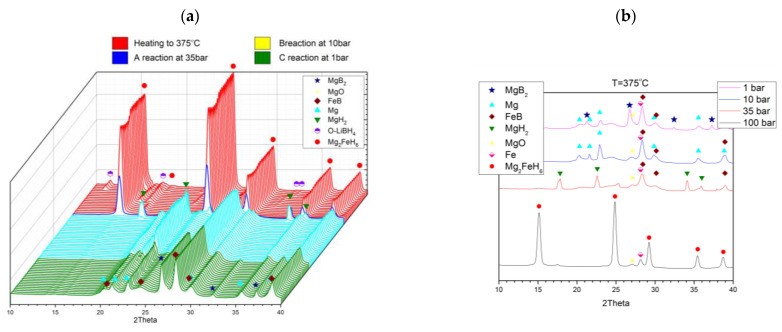
(**a**) In situ synchrotron XRD patterns of 2LiBH_4_–Mg_2_FeH_6_ undergoing dissociation at 375 °C at various H_2_ pressures. Measured at Lund (λ = 0.99242 Å). (**b**) In situ synchrotron XRD patterns taken just after the substeps of reactions A, B, and C at 375 °C.

The third decomposition scenario was carried out in isothermal mode at 375 °C (Figure 8a), serving to mimic the conditions for PCI (pressure–composition isotherm) dissociation. In this case, the sample was maintained in H_2_ at 100 bar, and heated to 375 °C. Over three steps, the pressure dropped progressively to 35, 10, and 1 bar, so as to witness and record the respective progress of the three A, B, and C substeps. Synchrotron XRD results (Figure 8a) emphasise that, for these prevailing conditions, substeps A, B, and C took place essentially independently, in full accord with earlier reported ex situ experiments and standard XRD studies reported in our previous work [11,26]. This finding was reinforced by scrutiny of the separate patterns taken in the wake of all substeps (Figure 8b).

### 2.3. Reaction C’s Lower Temperature Limit

**Figure 9:**
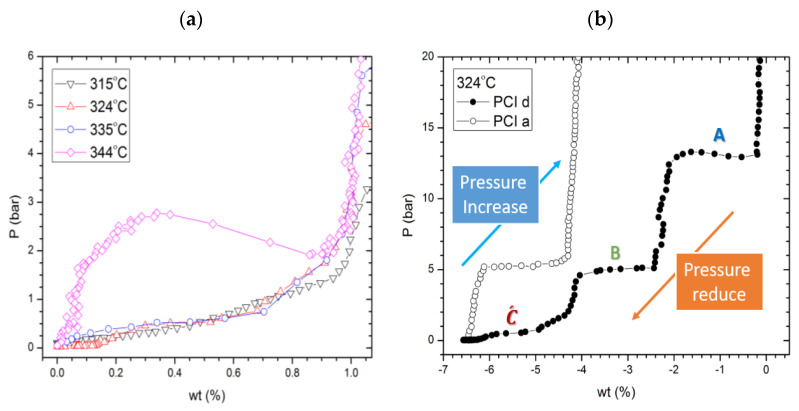
(**a**) PCI measurements of the three steps decomposition reaction at low temperatures. (**b**) PCI dehydrogenation/rehydrogenation cycle at 324 °C for reaction $\overset{´}{C}:\;2{\mathrm{LiBH}}_{4}\to \mathrm{LiH}+B+4H_{2}$.

PCI decomposition studies at various temperatures in the range of 315–344 °C reveal that the 2LiBH_4_–Mg_2_FeH_6_ decomposition reaction pathway alters at low temperatures (<340 °C); reactions A and B remain unaltered, while reaction C changed at lower temperatures. The plateau shape at temperatures below 340 °C was changed from a bumpy plateau to a lower pressure flat plateau (Figure 9a). The corresponding plateau pressure for multiple temperatures is mentioned in the presented van ’t Hoff plot (Figure 1), and discussed in detail in [11,27]. According to the presented van ’t Hoff plot, the theoretical pressure for known C reactions at 315, 324, and 335 °C ought to be between 2.1 and 2.7 bar (Figure 1), while the recorded plateau was near 0.5 bar. It is worth mentioning that the bumpy shape of the recorded plateau at 344 °C is the result of some instrumental/experimental limitations on the auto-detection of equilibrium points by our apparatus due to its slow kinetics. In a PCI run, the Sievert apparatus changes the pressure applied on the sample step by step, at a constant temperature. In a desorption study, if the observed pressure is lower than a defined threshold, the system will consider the observed pressure as a reaction point, and reduce the pressure for one more step; otherwise, it will wait for a longer time to either observe the required pressure drop or reach the maximum defined waiting time. As a result, at some points, where only the waiting time is satisfied, the recorded PCI curves are not in perfect equilibrium. For a better understanding of the details of how the employed Sievert apparatus operates and collects the PCI data, this is reported in the Supplementary Materials.

Moreover, to evaluate the products of dissociation at 324 °C, an isothermal rehydrogenation (PCIa) was accomplished on the same sample after a full dissociation reaction (Figure 9b). Here, the amount of absorbed H_2_ was approximately 50% in comparison with that which prevailed in the case of rehydrogenation at elevated temperatures. Based on the pressure of the plateau, this plateau can be considered to be the hallmark of hydrogenating pure magnesium (substep B). This indicates that at the new low-pressure plateau corresponding to sole LiBH_4_ decomposition ($\overset{´}{C}$) (Equation (5)), no MgB_2_ formed, and this corresponds to pure LiBH_4_ decomposition as the only hydrogen-containing compound remaining in the system.

## 3. Experimental Methods

Mg_2_FeH_6_ was mainly synthesized via two different methods: by ball milling in an inert gas atmosphere, followed by heat treatment under high hydrogen pressure (~100 bar) [21,28]; or by milling under reactive conditions, in a H_2_ or D_2_ atmosphere, with final treatment at low hydrogen pressure (~10 bar) [29]. A sample of 1.2 g of MgH_2_ and α-Fe (Sigma-Aldrich (St. Louis, United States), with a 2:1 molar ratio (52.0 wt% Fe), was put in a stainless steel vial with 10 of 10-mm-diameter stainless steel balls (ball-to-powder weight ratio = 30). This mixture was ball-milled in an argon atmosphere (1 bar) using a Retsch planetary ball mill (Haan, Germany) at 400 rpm for 30 h. The product was then transferred to an automated Sievert-style device (Advanced Materials Corporation) (Pittsburgh, USA) and sintered for 9 days at 400 °C under 100 bar of H_2_ pressure. The 2LiBH_4_–Mg_2_FeH_6_ composite was then prepared via mixing commercial LiBH_4_ (Sigma-Aldrich) powders with the synthesised Mg_2_FeH_6_ in a 2:1 molar ratio using an agate mortar. All mixing/treatment of the thus-produced samples was carried out in the glovebox in an Ar atmosphere.

### X-ray Diffraction

Ex situ XRD study was carried out using a Bruker D8 Advanced X-ray powder diffractometer (Cu K-α), using a secondary beam monochromator. To protect samples from air, a special holder was covered with Kapton film, which was placed above the diffraction plane in order to obviate any possibility of the polymer’s influence on the XRD patterns. In situ synchrotron radiation powder XRD (SRPXD) was carried out at the MAX-II Beamline I711 facility (Lund, Sweden). The high-resolution diffractometer uses Debye–Scherrer geometry with monochromators (λ = 0.99242 Å) and an MAR 165 CCD detector [30].

**Figure 10:**
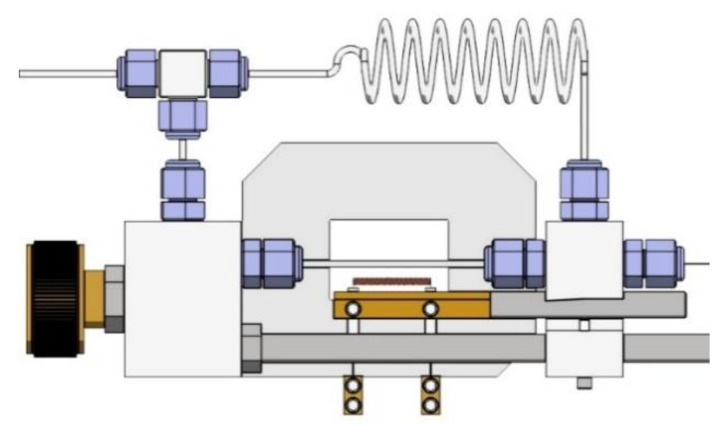
High-pressure holder (Aarhus, Denmark) for the samples for in situ synchrotron data measurement.

For in situ studies, a high-pressure sample holder with pressure adjustment options was employed (Figure 10) [31]. Loaded inside sapphire capillaries, the sample could be heated with a tungsten coil, controlled via an external PID regulator. The actual temperature of the sample was measured with the thermocouple placed in the powder bed. FIT2D (V 12.077) software was employed to record the area detector data, integrating peaks and exporting patterns in the format of 2θ-Intensity [32].

## 4. Conclusions

The dehydrogenation of the 2LiBH_4_–Mg_2_FeH_6_ assemblage was studied using in situ synchrotron X-ray diffraction techniques. It was found that this composite decomposes in two different paths depending on the applied conditions. Isothermal decomposition leads this composite to decompose in three different, essentially independent substeps: A, B, and C. Dynamic decomposition of this composite in the presence of hydrogen (10 bar) will cause the decomposition of MgH_2_ (reaction B) immediately after formation through reaction A (simultaneous A + B). Further heating of the products will end initiate reaction C and the formation of MgB_2_. Dynamic decomposition of the composite under vacuum serves to change the route of the final reaction, forcing LiBH_4_ to decompose alone (reaction $\overset{´}{C}:\;2{\mathrm{LiBH}}_{4}\to \mathrm{LiH}+B+4H_{2}$) without forming any MgB_2_, rather than the mixed decomposition of LiBH_4_ + Mg (reaction C). Kou et al. [33] attempted to rationalise the underpinning principles for the production of MgB_2_ under different decomposition conditions for the MgH_2_–LiBH_4_ system. The authors concluded that the formation of MgB_2_ consists of an incubation time, during which a period of growth of an initial nucleus was suggested. Their findings indicate that MgB_2_ formation is augmented by boosting the dehydrogenation pressure at the outset, at a constant temperature. Furthermore, it was observed that elevated temperature has the potential to reduce the waiting time. This phenomenon can easily explain why reaction C has a higher activation energy vis-à-vis pure LiBH4 decomposition and, therefore, higher temperatures are required for the initiation of the reaction.

Concerning the role of overpressure, according to the model we have suggested previously [12], the effect of pressure can be rationalised as a competition between two reactions: pure LiBH_4_ decomposition, and reaction C; indeed, both reactions are thermodynamically possible and viable. In a constant-temperature scenario, the larger overpressure (lower applied pressure) has a stronger effect on LiBH_4_ decomposition than reaction C; therefore, before the passage of the required incubation time in the case of reaction C, LiBH_4_ decomposition takes place exclusively.

In summary, three key points on the importance of solid–gas kinetic control parameters—such as overheating (T/Teq) and underpressure (P/Peq)—in the selection of favoured solid–gas reaction paths have been identified, which underpin the observed competition between reactions C and $\overset{´}{C}$:

- Longer incubation time required for reaction C;
- Lower overheating needed for reaction C (slower reaction);
- Lower underpressure (P-P_eq_) required for reaction C (slower reaction).

It is to be hoped that sophisticated molecular simulation, leveraging advances in density functional theory for predictive materials design, may be applied in future to allow for de novo metal hydride design and probe for even more efficient (semi-) catalytic material additives.

## Supplementary Materials

The following are available online. Figure S1: Gas Reaction Controller Sievert’s Apparatus layout, Figure S2: Non-equilibrium and equilibrium set points along a plateau.

## Sample Availability

Not available.
