# Supplementary material for: In Situ Synchrotron X-ray Diffraction Studies of Hydrogen-Desorption Properties of 2LiBH4–Mg2FeH6 Composite
Source: Molecules. 2021 Aug 11;26(16):4853. doi: 10.3390/molecules26164853 (PMC8398332; doi:10.3390/molecules26164853)
Supplement: Supplementary file 1 [file molecules-26-04853-s001.zip › molecules-1175373-supplementary.pdf]

Supplementary Information
file for

In-situ Synchrotron X-ray-Diffraction Studies of Hydrogen-DesorptionProperties of $2LiBH_4$-Mg_2FeH_6 Composites

Mohammad R. Ghaani[1,2*], Michele Catti[1], and Niall J. English[2*]

[1]Dipartimento di Scienza dei Materiali, Università di Milano Bicocca, via R. Cozzi 53, I-20125 Milano, Italy

[2]School of Chemical and Bioprocess Engineering, University College Dublin, Belfield, Dublin 4, Ireland

Pressure Composition Isotherm modes (PCI)

The GRC system's layout is illustrated in Fig. S1. The sample was placed inside "Sample CH1". The sample holder was an L-shaped stainless-steel tube placed inside a cylindrical furnace. It contained the sample holder - a little stainless-steel cylinder with internal volume of 4 cc and one thermocouple (TC1) placed exactly in the middle of the sample container; that sample holder connects to the internal part which was inside the main system box, and hosts the HP1 pressure transducer.

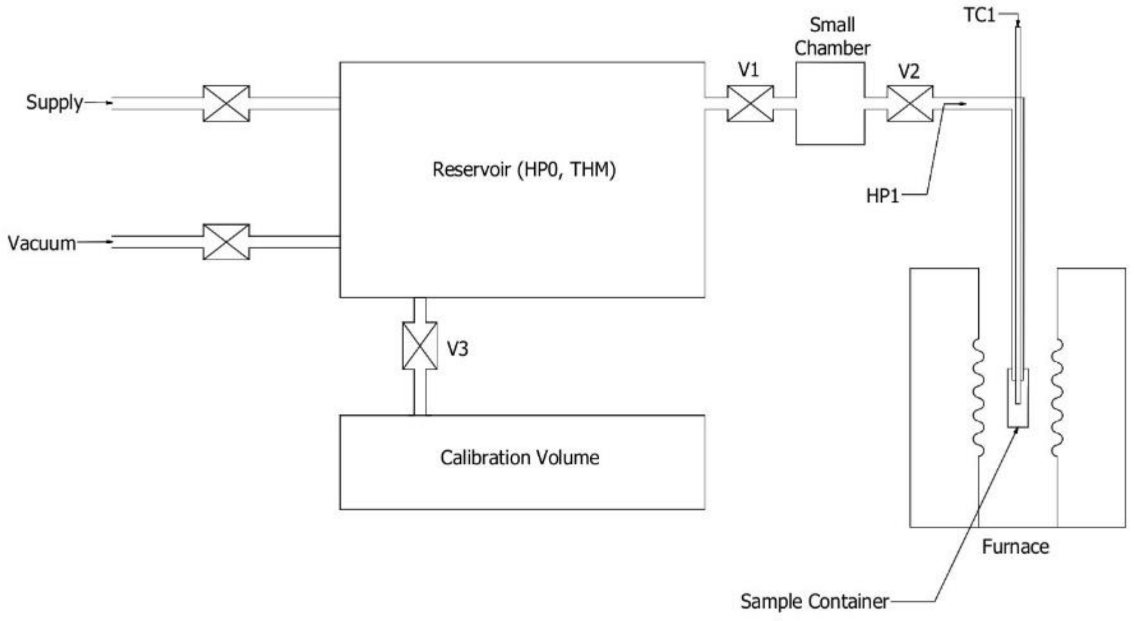

Figure S1. Gas Reaction Controller Sievert's Apparatus layout.

To measure the thermodynamic properties of hydrogenation/dehydrogenation reactions in isothermal condition, two PCI modes are available in this machine: 1- PCI Desorption Mode (PCId) and 2- PCI Absorption Mode (PCIa). With these modes, the system changed pressure on the sample step-by-step at a constant temperature. The system checked the experimental parameters every minute recording these "non-equilibrium data points" in a separate file (*.neq); if a reaction is taking place, the system observes a pressure (HP0) change in last 10 minutes "dp/dt"; this change is compared to the preset threshold dP: if

$$\frac{dp(HPO)}{dt} > \frac{dP}{10\ min} \qquad \text{Eq. 1}$$

the system considers that the reaction is still running and will not add or remove any gas until the settling condition is fulfilled, or it will wait for a certain period of time - the "Maximum Waiting Time, (t_{max})". When the settling condition is satisfied, the system records all the experimental parameters as "equilibrium point" and proceeds to the next target pressure. The measurement's primary terminating condition is reaching the specified final pressure (p_f) on the sample. The range of pressure settling condition is 0.1-0.01 bar; a low dP value increases

the resolution of the pressure plateau and is useful in case of reactions with slow kinetics. The total measurement time can be of the order of days.

The procedure of the PCI mode can be summarised as follows:

1. Reach the desired temperature at the starting pressure (p_0).
2. Set the dP and t_{max} values according to the reaction kinetics.
3. Set the final pressure (p_f) as terminating condition.
4. The system will calculate the step size (Δp) between subsequent points depending on $p_o - p_f$ and the selected resolution. These steps are not all equal for the whole PCI run; at higher pressures the step sizes are larger because of some technical design. This value can be changed during the run in the advance control panel, if required.
5. As one can see in Fig S2, for each non-equilibrium set of points the hydrogen release rate decreases, till the termination condition is fulfilled because the total pressure change in the last ten minutes (dp/dt) becomes smaller than $dP/10$, or the time spent for the set of points reaches t_{max}. The system considers the last point as the equilibrium point for the set, and it reduces the sample pressure by opening the V2 valve. The new pressure (p_s) is lower than that at the previous point, because the pressure in the small chamber before the V2 opening was lower.
6. During the run, every minute V1 and V3 open to equalise the pressure in the reservoir and the calibration and small chambers. This pressure value (HP0) is controlled by the system to apply the predetermined pressure difference (Δp) on the sample after the opening of V2.
7. In the next non-equilibrium set of points V1 and V3 open again, and the pressure of the small chamber is set back to HP0. In this case HP0 is slightly higher than its previous value.

Three types of plateau can be observed during PCI measurements:

- Nearly flat plateau (Fig. S2a):
    - The pressure difference between starting and final equilibrium points is about 2 bar.
    - This kind of plateau is usually observed when the reaction kinetics is fast and the dP condition is satisfied (Fig. S2a and S2b). For example, the decomposition plateaus of MgH_2 are roughly flat.

- The applied pressure difference at each point is roughly equal to that of the released hydrogen, so that the final equilibrium point for each set remains at a constant pressure (Fig. S2a, S2b).

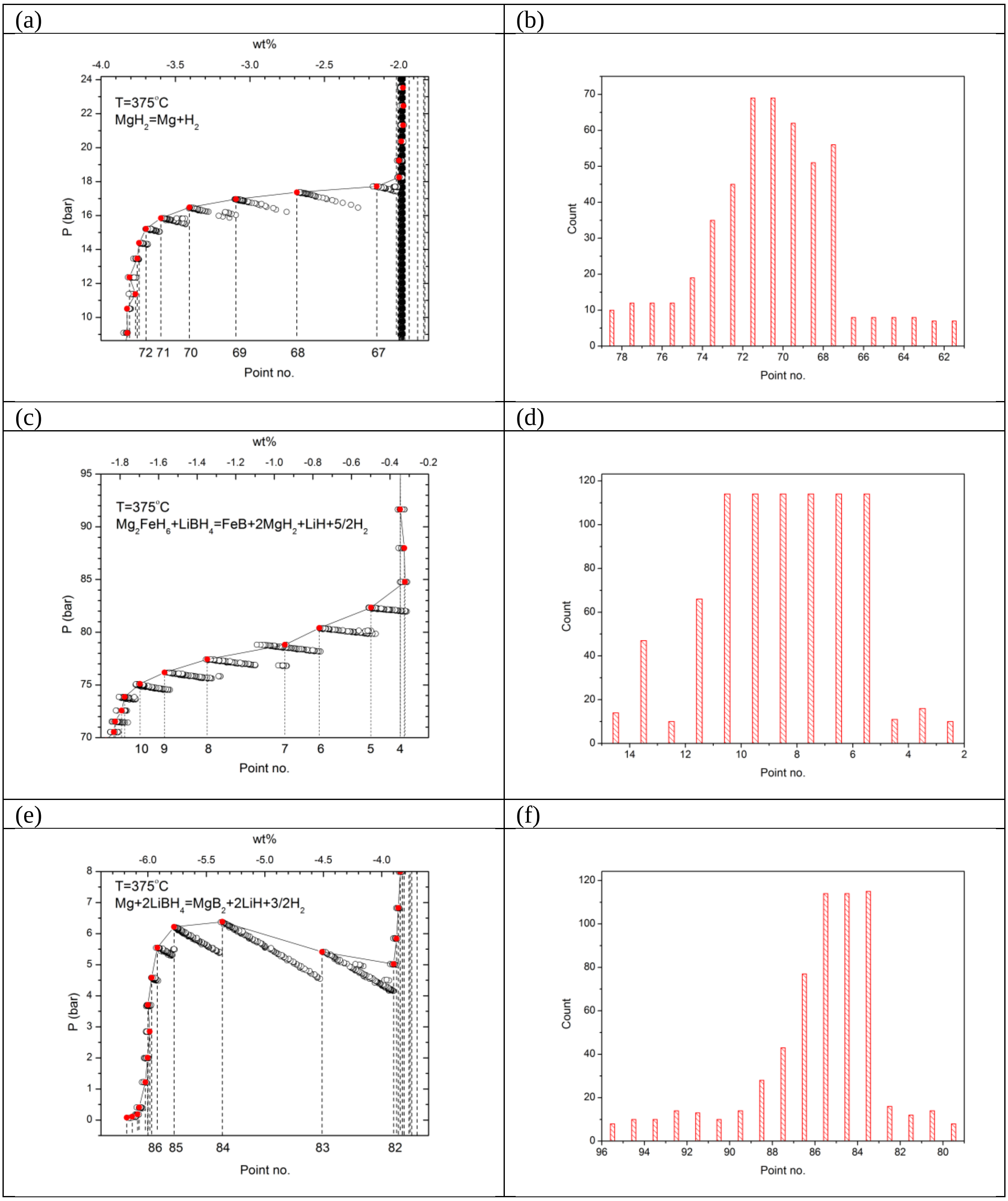

Figure S2. Non-equilibrium (open circles) and equilibrium (full circles) set points along a plateau (t_{max}=120min).

- Inclined plateau (Fig. S2c)
  - The pressure difference between starting and final equilibrium points may be about 10 bar.

    - In this case, the pressure of the released hydrogen at each point is much lower than the applied Δp. Reaching t_{max} always acts as a terminating condition. This occurs for reactions with slow kinetics. As it can be seen in Fig. S2c, S2d the number of non-equilibrium points is equal to t_{max} divided by the recording period time.
    - To have a better-defined plateau, one can change Δp manually to lower values or increase the maximum waiting time.
- ‘Bumpy’ plateau (Fig. S2e)
    - The situation observed is sometimes exactly opposite to that of the inclined plateau, *i.e.*, the applied Δp is low and the hydrogen release is large (Fig. S2e, S2f). Therefore the pressure of the equilibrium point of each non-equilibrium set is lower than that of the next equilibrium point. In this case, in the first half of the reaction, an inclined plateau with opposite slope will appear, but in the second part, the pressure of equilibrium points will decrease again.
